# Prediction of distal tibial articular extension in tibial shaft fractures: both posterior malleolar fracture and non posterior malleolar fracture intra-articular extension

**DOI:** 10.1007/s00068-022-02156-x

**Published:** 2022-12-14

**Authors:** Darren Myatt, Howard Stringer, James Chapman, Ben Fischer, Lyndon Mason

**Affiliations:** 1 Trauma and Orthopaedic Department, Liverpool Orthopaedic and Trauma Service, Liverpool University Hospitals NHS Foundation Trust, Liverpool, England; 2 University of Liverpool, Liverpool, England

**Keywords:** Tibial shaft, Tibial diaphysis, Posterior malleolar fracture, Intra-articular extension, Prediction, CT

## Abstract

**Background:**

Multiple authors have highlighted the increased incidence of occult posterior malleolar fractures (PMFs) with spiral tibial shaft fractures, although other reported associated risks of intra-articular extension have been limited. The aim of our study is to investigate both PMFs and non-PMFs intra-articular extensions associated with tibial diaphyseal fractures to try to determine any predictive factors.

**Methods:**

We undertook a retrospective review of a prospectively collected database. The inclusion criteria for this study were any patient who had sustained a diaphyseal tibial fracture, who had undergone surgery during the study period and who had also undergone a CT scan in addition to plain radiographs. The study time period for this study was between 01/01/2013 and 9/11/2021.

**Results:**

Out of 764 diaphyseal fractures identified, 442 met the inclusion criteria. A total of 107 patients had PMF extensions (24.21%), and a further 128 patients (28.96%) had intra-articular extensions that were not PMF’s. On multivariate analysis, spiral tibial fracture subtypes of the AO/OTA classification (OR 4.18, *p* < 0.001) and medial direction of tibial spiral from proximal to distal (OR 4.38, *p* < 0.001) were both significantly associated with PMF. Regarding intra-articular fractures, multivariate analysis showed significant associations with non-spiral (OR 4.83, *p* < 0.001) and distal (OR 15.32, *p* < 0.001) tibial fractures and fibular fractures that were oblique (OR 2.01, *p* = 0.019) and at the same level as tibia fracture (OR 1.83, *p* = 0.045) or no fracture of the fibular (OR 7.02, *p* < 0.001).

**Conclusion:**

In our study, distal tibial articular extension occurs in almost half of tibial shaft fractures. There are very few fracture patterns that are not associated with some type of intra-articular extension, and therefore, a low threshold for preoperative CT should be maintained.

**Level of evidence:**

4.

## Introduction

The reported incidence of diaphyseal tibial fractures is between 8.1 and 37.0/100,000/year [1–5]. Larsen et al. reported that AO-type 42-A1 was the most common fracture type, representing 34% of all tibial shaft fractures [1]. There are several studies which have demonstrated a high proportion of diaphyseal tibial fractures have ipsilateral occult posterior malleolus fractures (PMF), ranging from 22 to 92.3% [6–9]. These include several retrospective studies with small samples sizes, with variable diagnostic reporting explaining the high degree of variability in prevalence. Wang et al. published a systematic review on the incidence of missed diagnosis of occult PMF associated with tibial shaft fractures, finding that approximately 50% were missed on plain radiographs alone [10]. The presence of occult PMFs is of importance when treating tibial shaft fractures as there is an increased risk of intra-operative displacement if the tibia is fixed prior to the posterior malleolus [11].

Multiple authors have highlighted the increased incidence of occult PMFs with spiral tibial shaft fractures, although other reported associated risks of intra-articular extension have been limited [8, 10]. Hendrikx et al. have developed a machine learning predictive model to help highlight factors that may determine PMF associations with tibial shaft fractures [12]. PMF articular extension has received a reasonable degree of investigation; however, little is known about the non-PMF distal intra-articular extension and their predictive risk factors when associated with tibial diaphyseal fractures.

The aim of our study is to investigate both PMFs and non-PMFs associated with tibial diaphyseal fractures to try to determine any predictive factors taking into account both patient and injury characteristics. By identifying risk factors associated with PMF and non-PMF intra-articular fracture extension with a diaphyseal tibial fracture, we can hopefully direct the need for further radiological investigation, especially in systems of resource restriction.

## Methods

We undertook a retrospective review of a prospectively collected database. Data was collected on patients seen in the Liverpool University Hospitals Foundation Trust. The protocol was reviewed by the Liverpool Orthopaedic and Trauma Service research review board (Submission number 22-26) and was evaluated to be a service evaluation project and therefore, did not require ethical approval. Patients were identified using a prospectively stored patient record database (Bluespier international, Worcester, UK). All patients with diaphyseal tibial fractures who had undergone surgery were considered for this study. The time period for this study was between 01/01/2013 and 9/11/2021.

The inclusion criteria for this study were any patient who had sustained a diaphyseal tibial fracture, who had undergone surgery during the study period and who had also undergone a CT scan in addition to plain radiographs. The exclusion criteria were patients under the age of 16 or who had proximal extension of fracture into the knee joint. We reviewed the patients medical records, radiological imaging and collected demographic data. The patient records were reviewed with the use of digital imaging software (Vue PACS, Carestream, version 11.4.1.0324), Medway Sigma (Digital Health Intelligence Limited, London, UK) and Patient Electronic notes System (PENS).

**Fig. 1:**
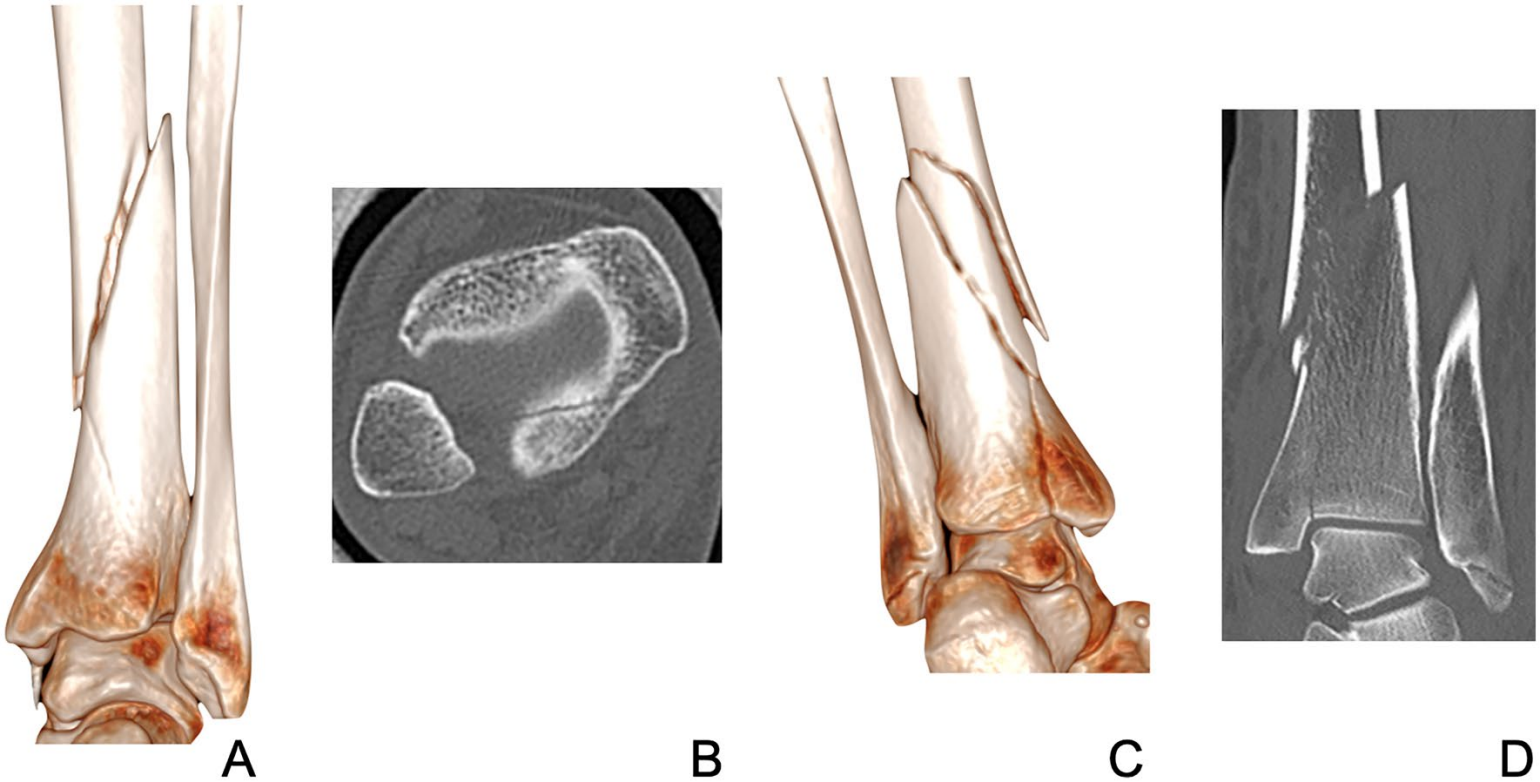
Illustration of 3D surface rendering (**A**) and axial CT scan (**B**) of a spiral shaft of tibia fracture with an undisplaced posterior malleolar fracture. In comparison, a 3D surface rendering (**C**) and axial CT scan (**D**) of a comminuted shaft of tibia fracture with a medial articular extension

The primary outcome of the study was to identify any factors that could predict distal articular extension in tibial shaft fractures (Fig. 1). Factors recorded included patient demographics, fibular fracture morphology, fibular fracture level, direction of tibial fracture extension, level of tibial fracture (using 1 Müller square from ankle articular surface to indicate distal fracture) and AO/OTA classification [13]. Two independent observers performed all radiographic observations including the categorisation by AO/OTA classification [13].

### Statistics

Continuous parametric data are presented as the mean ± standard deviation and dichotomous data as frequencies and percentages. Statistical analysis using the Cohen’s Kappa statistic for inter-rater agreement was performed, and inter-rater agreement was calculated. The inter-class correlation using intra-class correlation coefficient was interpreted according to Landis and Koch where slight agreement, 0.00–0.20, fair agreement 0.21–0.40, moderate agreement 0.41–0.60, substantial agreement 0.61–0.80 and almost perfect agreement greater than 0.81 [14]. For the reliability of intraclass correlation coefficient, confidence interval was set at 95%.

Uni- and multivariate analyses were performed using univariate and multivariate logistic regression analysis to identify independent predictors of distal intra-articular extension of tibial shaft fractures. The univariant analysis was performed using PMF extension and non-PMF extension as dichotomous dependent variables. Any factor which achieved significance on univariate analysis underwent further multivariant regression analysis. Significance was given to variables that reached *p* < 0.05. Statistical analysis was undertaken using SPSS statistics version 26 (IBM, New York, USA).

## Results

There were 764 diaphyseal fractures identified. Of these, 442 met the inclusion criteria. A total of 107 patients had PMF extensions (24.21%), and a further 128 patients (28.96%) had intra-articular extensions that were not PMF’s. Regarding the patients with PMF extensions, there were 64 males (59.81%) and 43 females (40.19%). The age range was 22–87 years (mean 45.99, SD 15.51). Regarding the patients with non-PMF articular extensions, there were 37 female (28.9%) and 91 male (71.1%). The age range was 19–82 years (mean 46.71, SD 16.28). When considering the AO/OTA classification, the Cohen’s Kappa statistic between the two reviewers was 0.747, demonstrating a substantial inter-rater agreement [13]. Of the PMF group, 81.04% (91/107) were undisplaced pre-surgery and in the non-PMF intra-articular extension group 36.72% (47/128) were undisplaced pre-surgery. In our study, two fractures displaced in surgery, one of which had not had PMF fixation and one that the PMF had undergone anteroposterior screw fixation.

**Fig. 2:**
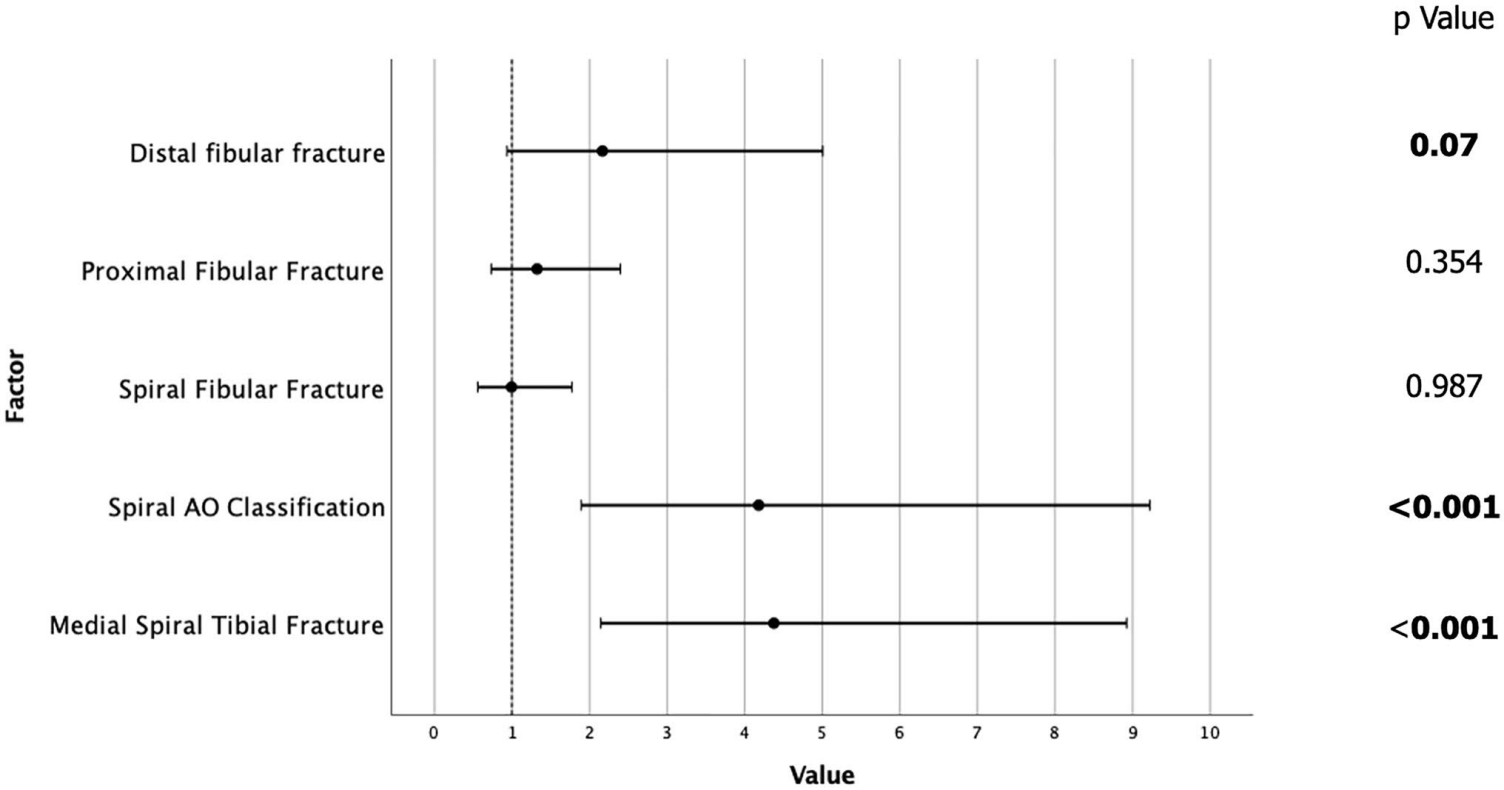
Multivariate analysis of factors associated with PMF extension of diaphyseal tibial fractures showing odds ratio (circle) and upper and lower bound error bars

The full univariate and multivariate analysis for PMF extension is displayed in Table 1. On univariant analysis, the PMF fracture extension was significantly associated with spiral tibial fracture subtypes of the AO/OTA classification (OR 12.01, *p* < 0.001), medial direction of tibial spiral from proximal to distal (OR 3.27, *p* < 0.001), and a fibular fracture which was proximal (OR 2.96, *p* < 0.001) or distal (OR 2.44, *p* = 0.013) and with a spiral morphology (OR 3.27, *p* < 0.001). On multivariant analysis, spiral tibia fracture morphology and medial direction of spiral remained significantly associated with PMF although fibular fracture location and morphology were no longer significant (Fig. 2). The multivariate model was 77.6% correct.

**Table 1:**
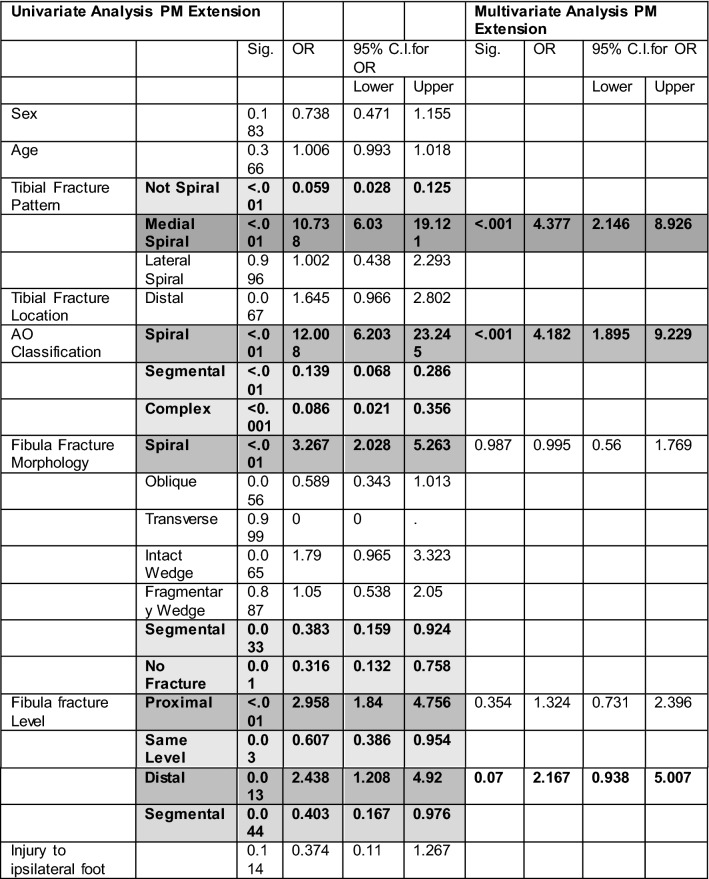
Univariate and multivariate analysis of PMF associated with diaphyseal tibia fractures

*OR* odds ratio, *Sig* significance, *CI* confidence intervals

Dark grey indicates positive significance, light grey indicates negative significance

**Fig. 3:**
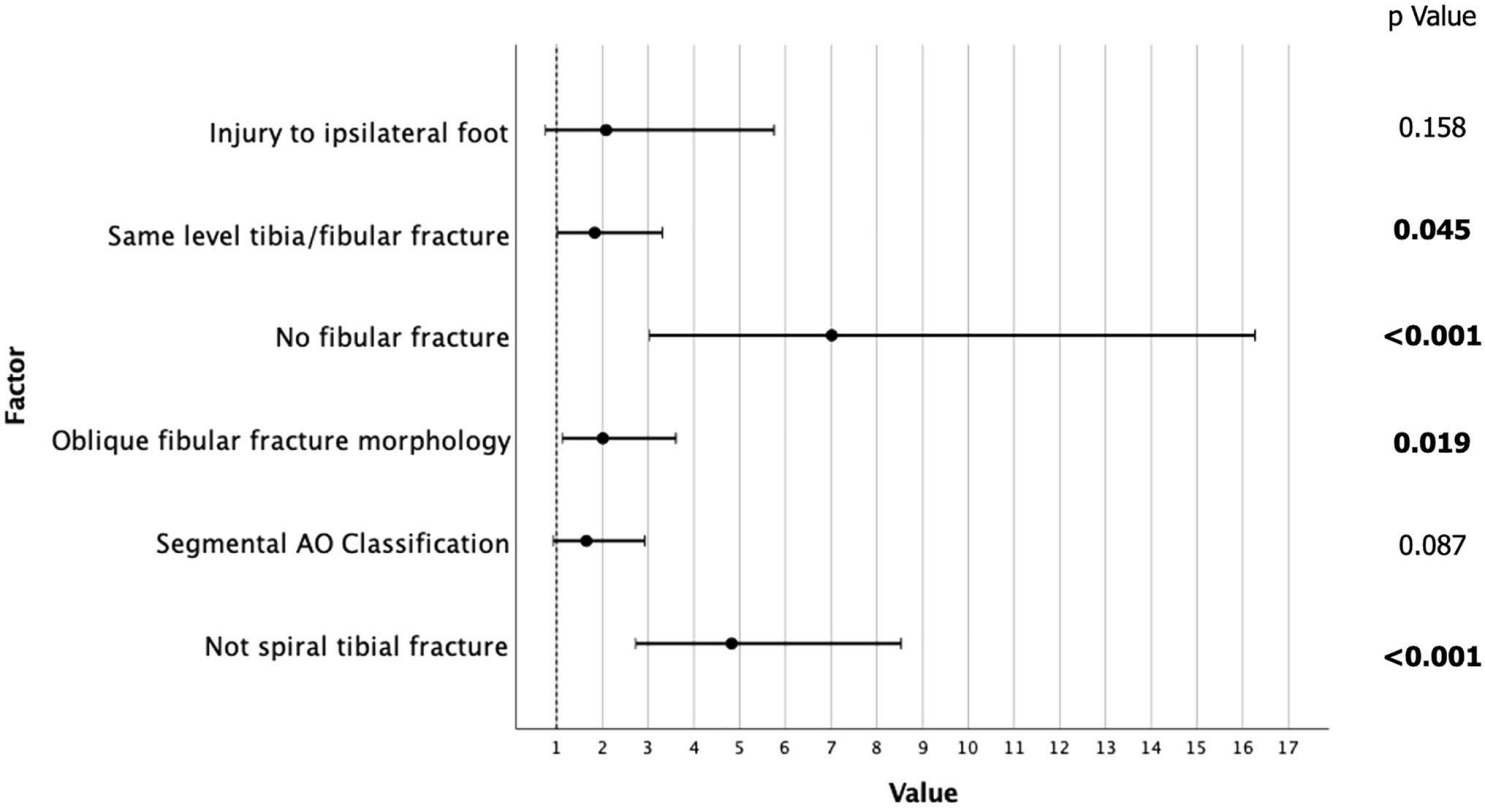
Multivariate analysis of factors associated with non-PMF intra-articular extension of diaphyseal tibial fractures showing odds ratio (circle) and upper and lower bound error bars. Distal tibial fracture was removed from this graph as the OR was too high to illustrate the other factors

The full univariate and multivariate analysis for non-PMF extension is displayed in Table 2. On univariant analysis for all intra-articular fractures that were not PMF showed that the significant associations were with distal (OR 6.1, *p* < 0.001) and segmental fractures (OR 2.75, *p* < 0.001) of the tibia. No fracture of the fibular (OR 3.84, *p* < 0.001), oblique fracture of fibular (OR 1.73, *p* = 0.017) or a fibular fracture at the same level (OR 2.59, *p* = 0.011) was also significantly associated. Injury to the ipsilateral foot was also a significant factor on univariate analysis (OR 5.54, *p* < 0.001). On multivariate analysis, both ipsilateral foot injury and segmental tibial fracture morphology lost significance (Fig. 3). The multivariate model was 83.3% correct.

**Table 2:**
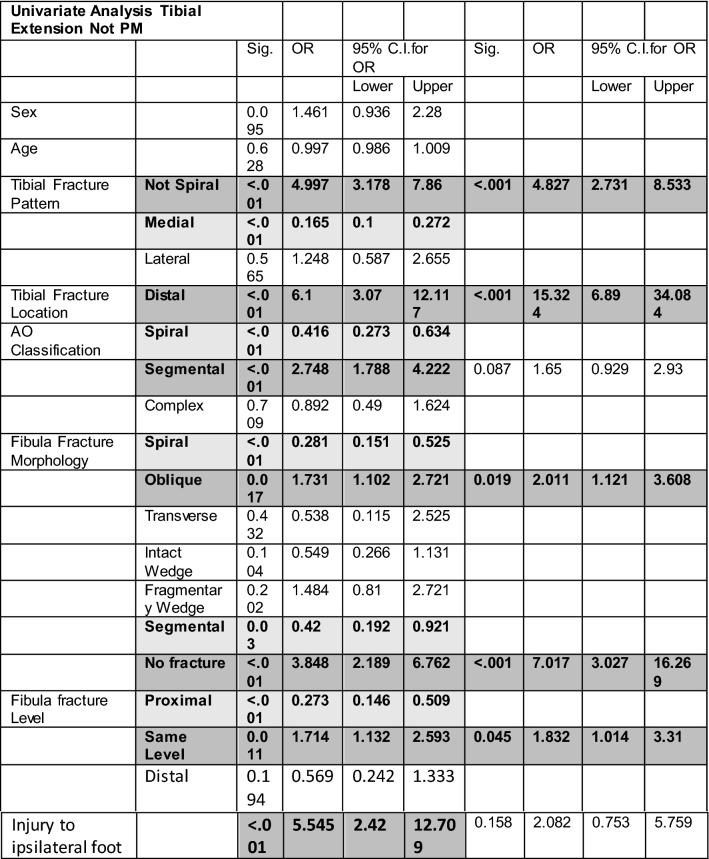
Univariate and multivariate analysis of non-PMF intra-articular extension associated with diaphyseal tibia fractures

*OR* odds ratio, *Sig* significance, *CI* confidence intervals

Dark grey indicates positive significance, light grey indicates negative significance

## Discussion

The primary outcome of the study was to identify any factors that could predict distal articular extension in tibial shaft fractures. We divided the types of distal articular extension into PMF and non-PMF fractures, which independently had different predictors on regression analysis of articular extension. On multivariate analysis, spiral tibial fracture subtypes of the AO/OTA classification and medial direction of tibial spiral from proximal to distal were both significantly associated with PMF. Regarding non-PMF intra-articular fractures, multivariate analysis showed significant associations with non-spiral and distal tibial fractures, fibular fractures that were oblique and at the same level as tibia fracture or no fracture of the fibular. 

For PMFs, many studies have previously illustrated a significant association with spiral tibial fractures [8, 11, 12, 15–18]. In our study, we agree that this was significant across all spiral tibial fractures on the AO/OTA classification. Our study has also demonstrated that the direction of the tibial spiral fracture was important, with medial proximal to distal direction being significant and lateral proximal to distal direction not. This finding has not previously been demonstrated in the literature. A number of authors also demonstrated that distal tibial fracture location was also significant for PMFs [8, 17, 18]. We did not find this in our study with PMFs, although there was both significance on univariate and multivariate analysis in the non-PMFs intra-articular extension group.

A number of factors were negatively significant for the association of PMFs and diaphyseal tibia fracture (i.e. their presence was significantly associated with the absence of a PMF), most commonly segmental fractures of both the tibia and fibular. The segmental tibia fracture was, however, significantly associated with non-PMF intra-articular fractures. Similarly, the spiral fractures were negatively associated with the non-PMF intra-articular fractures. This illustrates the difference in mechanism between the fracture types. In our opinion, the PMFs are almost certainly due to a rotational mechanism, likely from a proximal to distal direction due to the majority of the PMFs being undisplaced and therefore, the energy has dissipated finally through the PMF. In comparison, the non-PMF intra-articular extension however, is associated with complex and segmental fracture types, more in keeping with an axial load being applied to the foot with or without a varus or valgus force to the tibial diaphysis.

The prevalence of distal articular extension in tibial shaft fractures in our study was 53.17% (235/442). Of this, 45.53% (107/235) were PMF extensions and 54.47% (128/235) were non-PMF extensions. Considering the high occurrence rate of PMF and non-PMF intra-articular extension in tibial shaft fractures, a high index of suspicion should be kept. As illustrated by Larsen et al. the most common tibial fracture type is of spiral origin, and CT prior to surgery in these cases especially when a medial proximal to distal direction of tibial spiral fracture is present is in our opinion necessary if PMFs are to be observed [1]. Non-spiral fractures have a significant relation to non-PMF intra-articular fractures, and although the non-PMF intra-articular extensions are more commonly displaced, almost a third may be undisplaced and therefore, missed on plain radiographs. Therefore, there are very few fracture patterns that any degree of certainty could be given that a distal intra-articular extension was present.

With the high rate of occult fractures in the PMFs and non-PMF intra-articular groups, a low threshold for preoperative CT should be maintained. Jung et al. maintained that all spiral fractures of the tibia should undergo CT; however, this indication may need to be extended with the evidence of the non-PMF intra-articular fractures that this study provides [16]. Kempegowda et al. had previously illustrated a significant increase in intra-operative displacement in non-fixed PMFs and advocated a “malleolar first” protocol for tibial shaft treatment [11]. The number of intra-articular displacements in our study was low and occurred in both PMF fixation and non-fixation groups. We can therefore not add any further recommendation based on our findings.

The limitations to our paper are that it is a retrospective single centre review. A large number of tibial fractures did not undergo CT and therefore, were excluded. This therefore risks the under reporting of intra-articular extensions. This includes the susceptibility of some fracture patterns to have undergone CT based on the previous literature or surgeon anecdote and therefore, leading to some fracture patterns being over or under represented in the analysis. This study cannot comment on the long-term clinical outcomes of these injuries.

## Conclusion

In our study, distal tibial articular extension occurs in almost half of tibial shaft fractures. There are very few fracture patterns that are not associated with some type of intra-articular extension, when non-PMFs intra-articular extensions are also included. Therefore, a low threshold for preoperative CT should be maintained on all diaphyseal tibial fractures, especially in spiral and segmental fractures of the tibia.

## Data Availability

Data available on request from the author.
